# Psychological Growth and Post-Pandemic Stress Among Healthcare Professionals: Challenges and Opportunities

**DOI:** 10.1192/j.eurpsy.2025.1278

**Published:** 2025-08-26

**Authors:** M. Theodoratou, I. Skondra, C. Dafogianni, P. Andriopoulou, E. Denazi, V. Yotsidi, A. Kalaitzaki

**Affiliations:** 1 Social Sciences, Hellenic Open University, Patras; 2 Nursing, University of West Attica, Athens; 3 Psychology, University of Ioannina, Ioannina; 4 Psychology, University of Athens; 5 Psychology, Panteion University, Athens; 6 Social Work, Hellenic Mediterranean University, Herakleion, Greece

## Abstract

**Introduction:**

The COVID-19 pandemic placed an unprecedented burden on healthcare workers worldwide, particularly those in primary care settings, who faced both physical and emotional challenges.

**Objectives:**

The study aimed to assess the levels of psychological distress and anxiety, and the coping strategies among healthcare workers after the COVID-19 pandemic. It also examined changes in professional quality of life and the broader impact on personal life during the post-pandemic period.

**Methods:**

A cross-sectional study was conducted on a sample of 100 healthcare workers from various primary care centers in Patras, Greece. The following validated psychometric instruments were used:

- Secondary Traumatic Stress Scale (STSS): assessing symptoms of secondary traumatic stress, reflecting the emotional impact of indirect exposure to trauma through patient care.
- Quality of Professional Life Scale (5th edition): assessing both the positive (compassion satisfaction) and negative (burnout, secondary traumatic stress) dimensions of professional life.
- Posttraumatic Growth Inventory (PTGI): measuring positive psychological changes and personal growth experienced as a result of coping with pandemic stressors.
- Brief-COPE Scale: identifying coping strategies used by nurses, distinguishing between adaptive and maladaptive mechanisms.

**Results:**

The results of the study indicate a persistent and significant psychological distress among caregivers in the post-pandemic context of 2024. The STSS scores revealed elevated levels of secondary traumatic stress, underscoring the enduring emotional impact of the pandemic on healthcare workers. As demonstrated by the Quality of Professional Life Scale, a divergence in outcomes was observed. While a subset of nurses reported elevated levels of compassion satisfaction, a considerable proportion continued to exhibit high levels of burnout and secondary traumatic stress. This finding suggests that the adverse impact of the pandemic on professional well-being persists. The PTGI results revealed that some HCW experienced post-traumatic growth, suggesting the potential for positive psychological development despite the challenges encountered. Analysis of the Brief-COPE Scale depicted a diverse range of coping strategies, with evidence of both adaptive coping mechanisms, such as problem-focused strategies, and less adaptive responses, including avoidance behaviors.

**Conclusions:**

In conclusion, the results emphasize the persistent psychological impact of the ongoing pandemic on healthcare professionals, even in the post-pandemic era. These findings underscore the necessity for the provision of sustained psychological assistance and interventions that are specifically designed to address post-pandemic stressors, with the aim of ensuring the long-term well-being of healthcare workers.

**Disclosure of Interest:**

None Declared

